# Characteristics and trends in publication of scientific papers presented at the European Congress of Radiology: a comparison between 2000 and 2010

**DOI:** 10.1007/s13244-016-0511-8

**Published:** 2016-08-02

**Authors:** Will Loughborough, Helen Dale, James H. Wareham, Adam H. Youssef, Mark A. Rodrigues, Jonathan C. L. Rodrigues

**Affiliations:** 1 Bristol Royal Infirmary, University Hospitals Bristol NHS Foundation Trust, Upper Maudlin street, Bristol, BS2 8HW UK; 2 Sheffield Children’s Hospital, Sheffield, UK; 3 Southmead Hospital, Bristol, UK; 4 Centre for Brain Sciences, University of Edinburgh, Edinburgh, UK

**Keywords:** Abstracts, Publications, Research, Journal impact factor, Authorship

## Abstract

**Objective:**

To determine journal publication rates of scientific papers presented orally at the European Congress of Radiology (ECR) 2010, with comparison of country data to ECR 2000.

**Methods:**

All oral presentations from ECR 2010 were evaluated for publication between 2010 and 2014 using the MEDLINE database. Countries, collaborations, subspecialties, modalities and study design were ranked by publication percentage. Chi-square tests were used to compare publication percentages for each category of variables. Hazard ratios (HR) were calculated for each country relative to the host nation, Austria. ECR 2010 country statistics were compared with analogous data from ECR 2000.

**Results:**

In total, 360/840 abstracts were subsequently published (43 %). The author’s country of origin (*p* = 0.02), subspecialty (*p* = 0.02) and study design (*p* = 0.001) were significantly associated with subsequent publication. Switzerland, the Netherlands, France and Germany were among the top six countries by publication percentage in 2000 and 2010. In 2010, Switzerland had the highest publication rate (62 %) and HR in comparison to Austria (HR 2.62 [1.31–5.25], *p* = 0.01). Three Asian nations increased relative publication rates over the 10-year period.

**Conclusion:**

Several European nations consistently convert relatively high percentages of oral abstracts at ECR into publications, and the influence of Asian countries is increasing.

**Main Messages:**

• *Certain European nations consistently publish high percentages of orally presented abstracts at ECR.*

• *The influence of several Asian countries on ECR is increasing.*

• *Country, subspecialty and study design are significantly associated with journal publication.*

• *Authors collaborating internationally have the highest publication rates and mean impact factors.*

• *Among all modalities, PET-CT, MRI and CT have the highest publication percentages.*

## Introduction

Research helps to improve patient care, and as such is a pillar of clinical governance [1]. Radiology is a rapidly progressing speciality due to improvements in technology and the advent of new techniques. Evaluating these developments is essential in ensuring best practice in diagnosis and treatment. Therefore, assessing the quality of radiology research is important.

Scientific meetings allow practitioners to present and exchange ideas relevant to their field [2]. Although a certain standard must be reached for studies to be accepted as conference abstracts, only the most informative and highest-quality studies reach full publication in peer-reviewed journals [3]. Thus, the proportion of abstracts presented at medical meetings that are subsequently published in peer-reviewed journals can be regarded as marker of the research quality of the meeting and its participants [2]. In addition, the impact factor of the journal is a surrogate measure of the scientific quality of the articles published therein. It has been known since the 1950s that fewer than half of the papers presented at medical conferences are published as full-text articles [2]. Whilst the publication of abstracts from certain specialty specific conferences, such as orthopaedics and urology, have been explored in the literature [4–8], there is a paucity of data relating to journal publication rates and impact factors in radiology.

The purpose of this study was to evaluate journal publication rates and impact factors of oral abstracts presented at the 2010 European Congress of Radiology (ECR), the largest annual European radiology meeting, and one of the most influential radiology meetings worldwide. We aimed to assess the influence of the authors’ nationality and collaborations, as well as radiological subspecialty, modality and study design, on subsequent publication rates and impact factors. Additionally, we wanted to investigate temporal changes in publication rates and impact factors between ECR 2000 and 2010 to assess the changing contribution to radiological research by different countries.

## Materials and methods

All 840 oral abstracts presented at the 2010 ECR, as documented in the scientific sessions proceedings of the 22nd ECR, were retrospectively reviewed by three authors (WL, JW and AY) between September and December 2014. The following data were collected from each oral presentation: 1) country of first author, 2) level of author collaboration, 3) radiological subspecialty, 4) radiology modality, and 5) study design (prospective or retrospective). The MEDLINE database was searched by these authors to identify the oral abstracts from ECR that had been converted to full journal publications. Initial searches were performed with the first author’s name. If this failed to reveal a corresponding manuscript, further searches were performed with the other author names or appropriate keywords from the title. These search criteria were the same as those of an analogous study analysing publication rates from ECR 2000 [3], thus facilitating comparison. We replicated the follow-up period of 4 years, 9 months by selecting articles published between March 2010 (the month of ECR 2010) and December 2014. As in the previous study, only countries with ten or more publications were compared in the statistical analysis of country data. Only orally presented abstracts from ECR were included, and poster presentations were excluded. Authors were blinded to these statistics during data collection.

Any discrepancies were resolved by consensus with a fourth independent reviewer (JCLR).

The impact factor of each journal was obtained from the Science Citation Index on the Institute of Scientific Information (ISI) Web of Knowledge Journal Citation Reports [9] by a single independent author (MAR). The mean impact factor over the study period was calculated for each journal. These data were not available for the previous study.

## Statistical analysis

Authors’ countries of origin were ranked on a composite endpoint scale of percentage of abstracts published overall. Hazard ratios (with 95 % confidence interval [CI]) and *p* values) were calculated for each country relative to the host country, Austria. The previous study compared relative data to Austria with likelihood ratios [3]. Mean impact factor and publication rates per physician per 1000 population for the submitting country were calculated using World Bank data for 2000 and 2010 [10]. The two-tailed Fisher’s exact test was used to determine significance between datasets. Publication percentages and mean impact factors were calculated for institutional collaboration type, radiology subspecialty, modality and study design. These categories were then ranked on a composite endpoint score of publication rate. Chi-square tests were used to evaluate these variables for significant associations in publication rates. Two-tailed Fisher’s exact tests were used to determine significance in publication rates between subgroups of collaboration types and study design. All analyses were carried out using STATA version 12 statistical software (StataCorp LP, College Station, TX, USA).

## Results

At ECR 2010, there were 840 orally presented abstracts from 42 countries, of which 360 were subsequently published in the MEDLINE indexed full-text journal articles. This equates to a publication percentage of 43 %. There was no significant difference in publication percentage compared to ECR 2000 (ECR 2010: 360/840 vs. ECR 2000: 479/1020, *p* = 0.08). The mean IF for ECR 2010 publications was 3.35 (SD = 1.95); unfortunately, these data were not available for ECR 2000.

### Country of origin

The publication rate of abstracts was significantly associated with country of origin (X^2^ = 61.73, *p* = 0.02). Switzerland, Netherlands, France and Germany were consistently in the top six countries by publication rate in both 2000 and 2010 (Table 1). Switzerland had the highest publication rate (62 %) at ECR 2010 and the leading hazard ratio (HR) in comparison to Austria, the host nation (HR 2.62 (1.31–5.25), *p* = 0.01). Germany had the highest number of publications per physician per 1000 population in 2010 (27) and 2000 (58). The Netherlands had the highest mean IF (5.2) in 2010. These data were not available for ECR 2000. The publication rates of several Asian countries increased between 2000 and 2010: Japan (from unranked to 4th), South Korea (unranked to 5th) and China (unranked to 10th).

**Table 1:** Publication rates of presentations at European Congress of Radiology 2000^1^ and 2010 by author’s country of origin

	Country	Number of abstracts	Number of articles	Publication percentage	Relative likelihood	*p* value	Publications per physician per 1000 population	
2000								
	Austria	96	48	50	1.00	–	15.4	
	United States	21	16	76	1.52 (1.12–2.08)	0.03	6.2	
	The Netherlands	24	14	58	1.17 (0.79–1.73)	0.46	4.4	
	Germany	343	185	54	1.08 (0.86–1.35)	0.49	58	
	Switzerland	32	17	53	1.06 (0.73–1.56)	0.75	4.9	
	France	29	15	52	1.03 (0.69–1.55)	0.87	4.54	
	UK	76	38	50	1.00 (0.74–1.35)	1	20	
	Belgium^a^	29	14	48	0.97 (0.63–1.48)	0.87	3.6	
	Greece^a^	38	16	42	0.84 (0.55–1.29)	0.4	3.74	
	Italy	158	57	36	0.72 (0.54–0.96)	0.03	15	
	Other	174	59	34	0.68 (0.51–0.90)	0.01	-	
	Country	Number of abstracts	Number of articles	Publication percentage	Hazard ratios	*p* value	Publications per physician per 1000 population	Mean IF
2010								
	Austria^d^	36	12	33	–	–	2.5	3.5
	Switzerland^c^	39	24	62	2.62 (1.31–5.25)	0.01	5.8	3.7
	France^c^	38	23	61	2.33 (1.16–4.68)	0.02	6.8	4.3
	Netherlands^d^	46	27	59	2.28 (1.15–4.53)	0.02	9.3	5.2
	Japan^c^	21	11	52	1.77 (0.77–4.11)	0.18	4.8	3.1
	South Korea^c^	33	17	52	1.87 (0.89–3.91)	0.1	N/A^e^	3
	Germany^d^	222	101	46	1.52 (0.83–2.76)	0.17	27.3	3.2
	UK^d^	37	17	46	1.58 (0.75–3.31)	0.23	6.3	2.9
	Italy^c^	138	50	36	1.13 (0.60–2.12)	0.71	13.5	2.7
	United states^d^	35	13	37	1.17 (0.53–2.61)	0.69	5.4	3.7
	China^b^	52	18	35	1.15 (0.56–2.40)	0.02	12	2.4

^a^ At least 10 published abstracts in 2000 but not in 2010

^b^ At least 10 published abstracts in 2010 but not in 2000

^c^ Increase in publication rate rank in 2010 vs. 2000

^d^ Decrease in publication rate rank in 2010 vs. 2000

^e^ Physician per 1000 population data not available for South Korea for 2010

Hazard ratio, mean IF and citation data not available for ECR 2000

^1^2000 data taken from Miguel-Dasit A, Martí-Bonmatí L, Sanfeliu P, Aleixandre R (2006) Scientific papers presented at the European Congress of Radiology 2000: publication rates and characteristics during the period 2000–2004. European Radiology 16(2):445–450

### Collaboration

Collaboration between institutions was not significantly associated with subsequent publication (X^2^ = 16, *p* = 0.067). Authors collaborating internationally had a higher publication percentage (51 % vs. 44 %) (*p* = 0.35) and higher mean impact factor (3.2 vs. 3.1) than authors collaborating nationally (Table 2). International collaboration between a European country and the USA or a ‘rest of world’ country had the highest publication rate (67 %) (Table 3). National collaboration between institutions in the USA had the highest percentage of articles in the top quartile (50 %) and the highest mean IF, at 5.1.

**Table 2:** Rates of conversion of orally presented abstracts into publications according to local, national or international collaboration

Collaboration type	Abstracts presented	Abstracts published	Publication percentage	Top-quartile impact factor, *n* (%)	Mean impact factor	IQR
Local	609	253	41.5	60 (24)	2.6	1.97–3.68
National	131	57	43.5	14 (25)	3.1	2.42–3.68
International	97	49	50.5	15 (31)	3.2	2.05–3.94

**Table 3:** Rates of conversion of orally presented abstracts into publications according to collaboration subtypes

Institutional collaboration	Abstracts presented	Abstracts published	Publication percentage	Top-quartile impact factor, *n* (%)	Mean impact factor
Single European institution	494	209	43.18	52 (24.89)	3.34
Single US institution	13	3	23.08	1 (33.33)	3.17
Single ‘rest of world’ institution*	112	41	36.61	7 (17.07)	2.83
Two or more institutions in single European country	94	42	44.68	12 (28.57)	3.45
Two or more US institutions	9	4	44.44	2 (50.00)	5.06
Two or more institutions in single ‘rest of world’ country*	28	11	39.29	0 (0.00)	1.84
Different European countries	45	19	42.22	5 (26.32)	3.33
One or more European countries and USA	36	24	66.67	9 (37.50)	3.46
One or more European countries and ‘rest of world’ country*	6	4	66.67	1 (25.00)	3.03
Two or more non-European countries	10	2	20.00	0 (0.00)	2.42
Not specified	3	1	33.33		

*Rest of world country is any non-European/non-US country

### Subspecialty

Subspecialty was significantly associated with journal publication (X^2^ = 29.9, *p* = 0.01). Five subspecialties had publication rates over 50 %: paediatrics (63 %), thoracic (58 %), oncology (53 %), genitourinary (51 %) and MSK (50 %) (Table 4). ‘Computer studies’ had the lowest publication rate, at 17 %. ‘Safety issues’ had the highest number of published articles in the top-quartile impact factor journals, at 83 %, and a mean impact factor of 4.6. Gastrointestinal studies had the highest number of presentations (137) and publications (52), although its conversion rate was lower than that of other subspecialties (38 %).

**Table 4:** Publication by subspecialty

Subspecialty	Abstracts presented	Abstracts published	Publication percentage	Top-quartile impact factor, *n* (%)	Mean impact factor
Paediatrics	27	17	63	4 (24)	3.3
Thoracic	55	32	58	7 (22)	3.2
Oncology	32	17	53	3 (18)	3
Genitourinary	49	25	51	10 (40)	4
Musculoskeletal	70	35	50	10 (29)	3.5
Breast	54	26	48	0	2.7
Safety issues	13	6	46	5 (83)	4.6
Cardiac	84	37	44	12 (32)	3.5
Vascular	60	26	43	7 (27)	3.7
Neuroradiology	83	32	39	7 (22)	3.2
Gastrointestinal	137	52	38	16 (31)	3.4
Interventional	70	26	37	3 (12)	2.4
Head and neck	19	6	32	2 (33)	3.8
Physics	46	14	30	4 (29)	3
Quality improvement	10	3	30	0	1.2
Computer studies	29	5	17	0	1.8
Not specified	2	1	50	0	1.2
Total	840	360	43		

### Modality

There was no statistically significant association between modality and publication (X^2^ = 9.3, *p* = 0.23). PET-CT (48 %), MRI (47 %) and CT (43 %) had the highest publication rates (Table 5). Nuclear medicine (non-PET) had the lowest publication rate (25 %), although the one article that was published had the highest mean impact factor, with an IF of 6.2.

**Table 5:** Publication by modality

Modality	Abstracts presented	Abstracts published	Publication percentage	Top-quartile impact factor, *n* (%)	Mean impact factor
PET-CT	33	16	49	2 (13)	2.8
MRI	334	157	47	52 (33)	3.6
CT	259	112	43	27 (30)	3.3
Ultrasound	71	29	41	4 (14)	2.8
Fluoroscopy	69	23	33	3 (13)	2.6
Radiography	22	7	32	1 (14)	2.4
Mammography	25	7	28	0	3.7
Nuclear medicine (non-PET)	4	1	25	1 (100)	6.2
Not specified	23	8	35		
Total	840	360	43		

### Study design

Study design was the variable most significantly associated with subsequent publication (X^2^ = 12.2, *p* = 0.002). The mean impact factors of prospective and retrospective studies were comparable (3.5 and 3, respectively) (Table 6). Prospective and retrospective studies had similar publication rates (49 % vs. 48 %, respectively) (*p* = 0.99); however, the percentage of abstracts published in the top-quartile impact factor journals was higher in prospective studies (29 %) than in retrospective studies (19 %) (*p* = 0.08).

**Table 6:** Publication by study design

Study design	Abstracts presented	Abstracts published	Publication percentage	Top-quartile impact factor, *n* (%)	Mean impact factor
Prospective	289	142	49	41 (29)	3.5
Retrospective	117	57	49	11 (19)	3
Not specified	434	161	37	38 (24)	3.2
Total	840	360	43		

## Discussion

Our study reveals journal publication rates and journal impact factors of abstracts from an international radiology conference, according to author’s country of origin, over a significant length of time. Our results reveal that certain European nations consistently convert high proportions of abstracts into published manuscripts, whilst publication rates of several Asian countries (Japan, South Korea and China) have improved since 2000. We also demonstrate that the author’s country of origin, subspecialty, and study design are significantly associated with journal publication rates.

The 43 % publication rate in this study is in line with rates from non-radiological international medical conferences—for example, 21–47 % for urology conferences [4–6] and 34–50 % for orthopaedic meetings [7, 8]. Our data demonstrate marginally higher publication percentages than the limited data available from international radiology conferences (35–39 %) [11–13].

Our study methodology is analogous to that of a study of publication rates from ECR in 2000 [2], permitting a direct assessment of the temporal trend in publication rates of oral abstracts presented at a major international radiology conference. There was a non-significant difference in conversion rates of abstracts into publications, demonstrating consistency.

Comparison of the 2000 and 2010 data reveals that certain European nations, including Switzerland, France, the Netherlands and Germany, consistently convert high proportions of abstracts into publications. Interestingly, these four nations do not have the most densely populated radiologist populations in Europe [14, 15]. Similarly, only Switzerland among these four nations is in the top ten European countries by MRI or CT scanners per capita in 2010 [16]. The continued academic success of these countries therefore likely relates to greater promotion and investment in academic radiology than other European countries rather than overall number of radiologists or scanning capacity.

Some Asian countries, including China, Korea and Japan, increased the total number of publications, publication rates and overall ranking against other, predominantly European countries over the decade. These developments have occurred during a period of significant global economic change, and indicate the increasing globalisation of radiology research. Interestingly, over a similar time period, several major radiology journals have identified an increasing contribution of articles from Asian institutions, particularly in Japan and South Korea [17–20]. In contrast, decreases were observed in the USA and UK in both their absolute publication rate and ranking relative to other countries, which mirrors previous bibliometric studies [21], and may reflect the impact that an increasing clinical workload [22–24] and limited funding [25] has on academic radiology in these countries.

It is perhaps not unexpected that international collaboration between institutions produce abstracts that are more likely to lead to publication and higher impact factors than national or local authorship coalitions. Collaboration optimises existing resources and pools the expertise of leading academics across institutions. ECR 2010 took place after picture archiving and communication systems (PACS) had become a mainstream system globally. Along with high-speed Internet facilitating data transfer, this has broken down barriers that once made international collaboration more difficult. European nations are individually small, with relatively fewer research institutions and less funding [2] compared to countries such as the USA. Therefore, it is unsurprising that collaborations between European countries and either the USA or another ‘rest of world’ country have the highest abstract conversion rates. National collaborations between US institutions had the highest number of articles published in journals with top-quartile impact factors and highest mean impact factors, suggesting that the USA produces high-quality research.

Our subspecialty data demonstrate several trends comparable to those of other literature. The ECR 2000 study and another study analysing ECR 2001 [3, 26] also demonstrated high publication rates of thoracic- (56 and 49 %, respectively) and breast (55 and 53 %, respectively)-centred radiology research. Paediatrics has significantly improved from these two studies, currently converting 63 % of abstracts into publications (41 and 37 % previously). The high proportion of abdominal radiology abstracts and publications from our data is also reflected in the literature—one study analysing modalities represented in *Radiology* over a 10-year period [20] demonstrated abdominal-centred radiology studies to be the most frequent (1219/6542, 18 %), almost twice the number of any other subspecialty.

Our modality data are in line with those of other studies in recent years with regard to MRI and CT being the most prevalent modalities in published radiology research. One study demonstrated that between 2001 and 2010, original articles on MRI and CT were by far the most frequent modalities in major radiology journals (30 and 27 %, respectively) [20]. A separate study analysing modalities from manuscripts published in *AJR* and *Radiology* in 2001 found that MRI was the major modality in 31 % and CT in 27 % of journals [18]. The high publication rate of PET-CT in our more recently acquired data highlights the emergence and significance of this modality in more recent years.

Whilst prospective and retrospective studies have similar rates of conversion into published manuscripts, our results suggest that high-impact-factor journals consider prospective studies more favourably. A study examining acceptance rates to a major European cardiology conference also found prospective non-randomised study design to be an independent predictor of acceptance [27].

Our study had limitations. Firstly, we determined publication status through a MEDLINE search. As MEDLINE focuses on English language journals, articles not published in English will be underrepresented [2, 28]. However, we consider the MEDLINE database as currently the largest available database of relevant medical and radiology abstracts. Additionally, MEDLINE acts as a further quality control measure, as a committee selects journals for inclusion on the basis of their scientific policy and quality.

The key surrogate marker of research quality in our observational study was the percentage of abstracts published in peer-reviewed journals. An important consideration, therefore, is that of publication bias, which is where studies with statistically significant results are more likely to be published, and published earlier, than studies with no significant difference between study populations [29, 30]. Our initial study population of all oral abstracts at ECR 2010 had already undergone peer review prior to acceptance into the conference, and was thus potentially subjected to publication bias. To effectively analyse publication bias in the context of our study, all accepted and rejected ECR abstracts would need to be included and analysed. A further key surrogate indicator for research quality in our study was the impact factors of the publishing journals, a marker of the average number of citations [31]. This is not without fault, and several variables may influence the number of citations, such as the language of the publishing journal and the domain concerned [32]. It is estimated that the top 20 % of abstracts generally receive 80 % of journal citations [33]. High-impact-factor journal publication thus does not directly translate into an article receiving a high number of citations. However, in our study, we considered impact factors as a suitable objective quality assessment with which to evaluate a large number of abstracts. Additionally, the use of a normalised indicator such as percentage of articles published in the top quartile of impact factors allowed better comparison [2].

We indexed publication rates to number of doctors per population to determine the countries with the highest publication capacity. A more useful comparison would have been indexing to the number of radiologists. However, the most recent available data on this are from 2006 [14], and we therefore felt that indexing publication rates to concentration of doctors overall for 2000 and 2010 was more reliable.

A potential bias in the assessment of collaboration is that the author’s affiliation in the ECR book of abstracts may not be a true representation of the country in which the majority of the project was performed. For example, authors move around during their residency and may submit an abstract with an affiliation to a former institution, whilst the work was performed at their current institution [2].

Finally, we compared data from two points in time: 2000 and 2010. It is possible that the trends identified may represent an anomaly rather than a linear progression. Further studies are needed from future ECR meetings to consolidate our findings, enabling insight into the leading global radiology researchers of the future.

## Conclusion

Our study demonstrates interesting characteristics and trends in the publication of radiology research between ECR 2000 and ECR 2010. At ECR 2010, the author’s country of origin, subspecialty and study design were significantly associated with subsequent publication, whilst author collaboration and modality were not. Certain European nations consistently convert relatively high numbers of presented abstracts into published manuscripts, and an increase among certain Asian nations has occurred over the 10-year period.
